# Stability of the Meat Protein Type I Collagen: Influence of pH, Ionic Strength, and Phenolic Antioxidant

**DOI:** 10.3390/foods9040480

**Published:** 2020-04-11

**Authors:** Massimo Lucarini, Alessandra Durazzo, Fabio Sciubba, Maria Enrica Di Cocco, Raffaella Gianferri, Mosè Alise, Antonello Santini, Maurizio Delfini, Ginevra Lombardi-Boccia

**Affiliations:** 1 CREA-Research Centre for Food and Nutrition, Via Ardeatina 546, 00178 Rome, Italy; alessandra.durazzo@crea.gov.it (A.D.); g.lombardiboccia@crea.gov.it (G.L.-B.); 2 Department of Chemistry, “Sapienza” University of Rome, Piazzale Aldo Moro 5, 00185 Roma, Italy; Fabio.sciubba@uniroma1.it (F.S.); mariaenrica.dicocco@uniroma1.it (M.E.D.C.); raffaella.gianferri@uniroma1.it (R.G.); maurizio.delfini@uniroma1.it (M.D.); 3 Department of Pharmacy, University of Napoli Federico II, Via D. Montesano 49, 80131 Napoli, Italy; alisemose@gmail.com (M.A.); asantini@unina.it (A.S.)

**Keywords:** meat, collagen, pH, ionic strength, catechin, ^1^H NMR, T_2_ relaxation

## Abstract

The water-holding capacity (WHC) is among the key factors in determining the quality of meat and its value, which is strongly influenced by the content and quality of the connective tissue proteins like collagen. Therefore, the factors that influence the proteins’ stability, e.g., pH, ionic strength, and the antioxidants which are used to increase the meat shelf-life, also affect the WHC. The interaction of collagen, whose structure is strongly influenced by the interaction with water molecules, can be studied following the behavior of water diffusion by low-resolution ^1^H NMR experiments. The present study is addressed to study the collagen stability as a function of pH, ionic strength, and phenolic antioxidants like catechin. The experimental study demonstrated how the ^1^H NMR time domain (TD) experiments are able to evaluate the hydration properties of collagen, not only as a function of ionic strength and pH, but also in determining the ability of catechin to interact both on the surface of the collagen fibrils and inside the fibrillar domain.

## 1. Introduction

The water content of meat, approximately 75%, and the water-holding capacity (WHC) are both key factors in determining the technological quality of meat and that of meat products [1,2].

Among the constituents of meat, proteins play a fundamental role in retaining water, [3] and, consequently, the factors influencing the protein stability also affect the WHC [1]. The main proteins responsible for the WHC in meat are myofibrils and the collagen of connective tissue. Myofibrillar proteins are the most studied, since they are responsible for most of the water retained in the muscles. Collagen is ubiquitous in all vertebrates, and its structure is stabilized by extensive hydrogen bonds where water molecules are a relevant part of the ordered hydrogen-bonding existing network [4]. Among the factors to be considered in the technological processes of the conversion of muscle into meat, there are the pH, the ionic strength, and the antioxidant molecules, which are used to slow down the oxidative process onset.

Changes in the pH around the isoelectric point of the meat proteins causes a change in the net charge (positive or negative) of proteins, determining a greater electrostatic repulsive force among the protein chains, which leads to an increased swelling of the protein system with an increase in the WHC. Similarly, the meat proteins’ electronic charge is also affected by the ionic strength that is able to move the isoelectric point of the proteins, causing a different mobility of the water inside the protein domain system. Oxidation of meat has a relevant effect on quality parameters, and for this reason, antioxidant molecules are used to slow down this process. The use of natural antioxidants to prevent and slow down the oxidation process in biological matrices is relevant. Proteins in biological matrices are, together with lipids, one of the main targets of the oxidant compounds for both their reaction rate and abundance [5,6]. 

Recently, a mechanism for the antioxidant action of catechin, a flavan-3-ol, namely (2R, 3S)-2-(3,4-dihydroxyphenyl)-3,4-dihydro-2H-chromene-3,5,7-triol, towards collagen, has been proposed [7]. Madhan et al. [8], by using circular dichroism and Fourier transform infrared spectroscopy (FTIR), reported how hydrogen bonding and hydrophobic interactions represent the main forces in collagen stabilization due to the interaction with vegetal origin polyphenols. The interaction of catechin with the collagen could influence the WHC of the macromolecule; in fact, the molecular size of catechin fits the ones of collagen pentafibrils. These last can then stabilize the collagen structure through inter and intramolecular hydrogen bonds [8]. Collagen molecules can differ, not only in their molecular and supramolecular structures, but also in their distributions in the tissues and their functions [9]. In this study, attention has been mainly addressed to type I collagen, since it is ubiquitous in all vertebrates, and it represents at least 90% of total collagen types [10].

Nuclear magnetic resonance time domain (NMR TD) is a nondestructive method that allows the characterization of water mobility in addition to its distribution [11]. Many studies published have correlated low-field ^1^H NMR measurements with the WHC. The transverse relaxation time (T_2_) has been used to get information on the meat structure, with a high correlation with meat proteins’ properties [12]. Low-resolution ^1^H NMR has been used to characterize foods by determining both the decay rate and the amplitude of the ^1^H NMR signal. The T_2_ relaxation curve in heterogeneous systems is represented by a multiexponential decay curve that can be attributed to the presence in the sample of elements with structural differences [13]. The decay rate of the ^1^H NMR signal is lower in these systems compared to the one corresponding to bulk water and can be quantitatively rationalized. In this study, the dynamical properties of the water molecules present in the hydration network of collagen by a systematic approach based on ^1^H NMR signal relaxation determinations have been assessed. The behaviors of water dynamics under different experimental conditions that can occur in meat products have been investigated by evaluating the water proton relaxation rates. Physiological changes in the ionic strength and pH have been considered, as well as the effects of the interactions of collagen with a small antioxidant molecule like catechin. Further information about collagen and water dynamic behaviors have been obtained by transverse ^1^H NMR relaxation times and self-diffusion measurements. To the authors’ knowledge, no other study has been up to today reported on the use of ^1^H NMR TD to assess the functional properties of collagen.

## 2. Materials and Methods 

Collagen type I fibers from bovine Achilles tendon (BAT), (±) catechin and all reagents were purchased from Sigma (Sigma Chemicals Co., St. Louise, MI, USA) and were used without further purification for the experimental study. 

### 2.1. Study of Collagen Stability as Function of pH and Ionic Strength

Three different solutions were prepared at pH 5.4, 6.2, and 7.0 and each of them with three different ionic strengths (0.29, 0.45, and 0.71 M), for a total of nine buffer solutions.

The range of pH and ionic strengths have been chosen and considered in order to represent the variation of these parameters in the meat [14]. The buffer solutions were prepared using 50 mM citric acid monohydrate (10.507 g/L) and trisodium dehydrate (14.707 g/L). pH was adjusted with 4 M NaOH or 4 M HCl, and ionic strength (I) was adjusted by the addition of NaCl according to the following Equations (1):

(1) $$I=\frac{1}{2}\sum^{\;}CiZi^{2}$$

where *Ci* stands for the concentration, and *Zi* stands for the ion charge. Considering our experiment, the ion strength (I) was calculated by using the following Equation (2):

(2) $$I=\frac{1}{2}\left([H_{2}A\right]1+\left[HA^{-2}\right]2^{2}+\left[A^{-3}\right]3^{2}+2\left[NaCl\right])$$

Collagen was hydrated by adding a buffer solution to dried samples and left for two days at 4 °C before ^1^H NMR experimental determinations. 

### 2.2. Catechin-Treated Collagen

Collagen (2.5 mg/mL) was treated with catechin (0.001 M) for 24 h at room temperature (25 °C) in the dark and without any shaking, as described in the supplementary material of a previously published work by Lucarini et al. [7]. Catechin solutions were spectrophotometrically titrated at 280 nm using a Cary 60 UV–Vis spectrophotometer (Agilent Technologies, Santa Clara, CA, USA) before and after the collagen addition to estimate the catechin amount in the complex. 

Collagen-catechin complex sample was dialyzed for 24 h at 4 °C against distilled water. Dialysis bag, manufactured by Spectrum Medical Industries and with a molecular weight cut-off less than 14KD, were purchased from Fischer Scientific (Waltham, MA, USA). At the end of dialysis, the collagen-treated samples were freeze-dried. After catechin treatment, the collagen color became slightly pink.

### 2.3. ^1^H NMR Time Domain (TD) 

Samples were hydrated by an addition of deionized distilled water or one of the nine buffer solutions and left at 4 °C for two days to allow collagen hydration. The ratio buffer and dry collagen matter was 3 (*g*/*g*). The hydrated samples were placed in ^1^H NMR tubes and immediately sealed. 

Measurements were performed with a Minispec mq 20-pulsed ^1^H NMR spectrometer (Bruker Spectrospin Company, Silberstreifen, Germany) with an operating frequency of 20 MHz for protons (magnetic field strength: 0.47 T). The NMR spectrometer was equipped with an external thermostat (Julabo F25, Julabo Labortechnik GmbH, Seelbach, Germany) in order to maintain the selected temperature conditions (t = 25 °C). Before the NMR measurements, the tube was placed into the ^1^H NMR probe as long as needed for thermal equilibration (t = 15 min).

Longitudinal relaxation measurements (T_1_) were performed by an inversion recovery (IR) sequence.

Transverse relaxation measurements (T_2_) were performed by a Carr-Purcell-Meiboom-Gill (CPMG) sequence [15]. For each sample, 49 scans were acquired, with a recycle delay of 40 s. The decay of the transverse magnetization was triexponential; the amplitudes and relaxation rates of the three components were calculated by a nonlinear least-squares data fitting with a proprietary self-made computer software based on the Marquardt algorithm [16].

Self-diffusion measurements were carried out using a standard Stejskal-Tanner (PFG spin-echo) sequence [17]. 

## 3. Results

### 3.1. ^1^H NMR Characterization of Type I Collagen

#### 3.1.1. Time Domain Measurements

The water properties of collagen recovered from the bovine Achilles tendon system were studied by ^1^H NMR T_2_ relaxation measurements.

The CPMG decay data found for hydrated collagen samples were multicomponent, as generally reported in compartmentalized or heterogeneous systems [18,19]. The sum of three exponentials gave the best fit. As shown in Table 1, the three T_2_ values were 6 ± 1, 41 ± 7, and 447 ± 15 ms, with relative fractions of 56 ± 5, 36 ± 4, and 8 ± 2%, respectively.

Table 1 shows the three values of Di associated with the three water fractions. The Di values were D_1_ = 0.71 × 10^−5^ cm^2^ s^−1^ for the fast-relaxing fraction 1, D_2_ = 1.22 × 10^−5^ cm^2^ s^−1^ for the intermediate fraction 2, and D_3_ = 2.17 × 10^−5^ cm^2^ s^−1^ for the slow-relaxing fraction 3. We observed from relaxation measurements in hydrated collagen that multiple water species were detectable (the water relaxation curve was three exponential), with one component having the same relaxation time as pure water and the others two characterized by a reduced T_2_. The three water fraction localizations were assigned based on available literature data [18,19,20]. Considering the work by Kopp et al. [21], they describe a protein solution which can be viewed as made by three different water environments: the buried water molecules, the water hydration shell around the protein, and the bulk water [21].

In particular, the structural bonded water corresponds to unfreezable water molecules in line with the collagen hydration models proposed [21,22]. They are linked by triple hydrogen bonds involving hydroxyproline or by double hydrogen bonds in the available sites inside the triple helix [22]. It has been supposed, assuming non-interacting bonds, that four hydrogen bonds are involved in the linkage of water at the protein–water interface, as it has been reported by Privalov [4]. 

A cross-relaxation and transverse relaxation ^1^H NMR study of partially hydrated collagen (h < 0.3), at different temperatures and water activities, showed that T_2_ values of water protons were less than 300 ms [23]. In the experimental conditions of this research (CPMG echo time of 100 ms), the ^1^H NMR signal cannot be assigned to this interstitial water fraction, whereas the water molecules localized in the micro fibrils (between the triple helix of tropocollagen) mainly contribute to the shortest transverse relaxation time (T_21_ = 6 ms). The intermediate T_2_ (T_22_ = 41 ms) was assigned to the water fraction placed in the interfibrillar space. A part of this water fraction is related to the collagen structure and provides water bridges between the collagen microfibrils [24].

The molecules of water present in fraction 2 are more mobile than those of fraction 1 but are affected by the macromolecule, as confirmed by the small differences in the related diffusion coefficients (D_1_ = 0.71 × 10^−5^ cm^2^/s and D_2_ = 1.22 × 10^−5^ cm^2^/s). These two slow-relaxing fractions are the hydration water of the tropocollagen. The phenomenon of restriction explains their D_1,2_ values, whereas the observed decrease is due to the so-called direct hydration effect [25].

For the third fraction, a long T_2_ (T_23_ = 447 ms) is reported, as well as a diffusion coefficient (D_3_ = 2.31 × 10^−5^ cm^2^/s) close to the value for pure water, and corresponds to the “free” or “bulk” water fraction.

The best fit obtained for the longitudinal relaxation time T_1_ was monoexponential, with a value of 950 ms. The ratio T_1_/T_2_ was found >1.6. This result indicated that the motion of water molecules was anisotropic and characterized by a distribution of correlation times [26].

That T_1_ was monoexponential is not anomalous, since in heterogeneous macromolecular systems, T_2_ is almost exclusively considered. This is related to some experimental aspects in the measurement of relaxation times: while T_2_ can be carefully and quickly determined by the CPMG sequence, an accurate T_1_ determination can be challenging in heterogeneous systems, where, generally, T_1_ >> T_2_. Since measuring the recovery curve of the longitudinal magnetization using the inversion recovery sequence requires an adequate series of recovery delays and long repetition times (≥5 T_1_), to ensure complete longitudinal relaxation, a number has often been measured low of the experimental points for T_1_ determination, and it has not always been possible, by deconvolution of the curve, to calculate the same number of T_1_ components as those obtained from T_2_ measurements. Anyhow, the correspondence of the T_1_ values with the T_2_ values and the ratio T_1_/T_2_ can be considered sufficient for the data interpretation, since this ratio is potentially useful information for the assessment of the anisotropy existing in heterogeneous systems [13]. 

**Figure 1:**
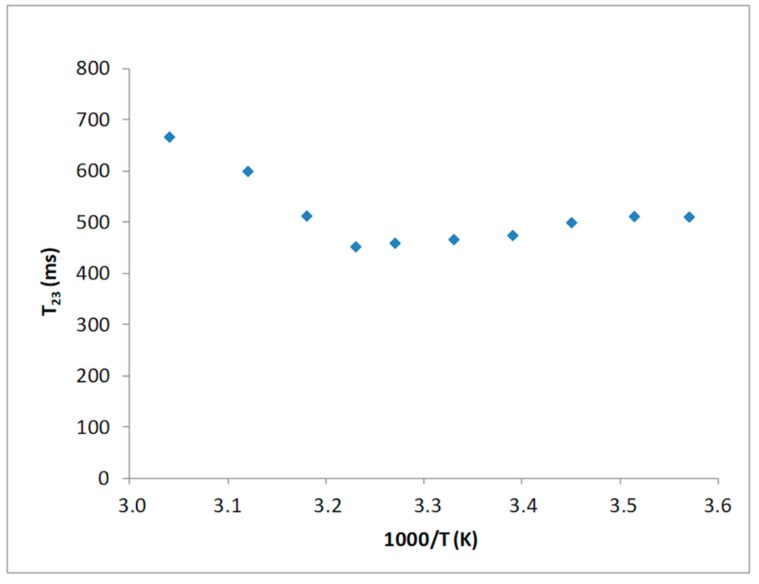
Temperature dependence of the T_23_ values of the collagen sample.

Several works reported for water molecules not undergoing proton chemicals exchange a linear relationship of the proton spin-spin relaxation time with the reciprocal of the temperature [27,28]. Indeed, a T_2_ minimum is expected if the chemical exchange mechanism is the main contribution to relaxation. In a temperature range from 277 to 323 K, such a behavior has been displayed by the T_23_ component, as reported in Figure 1.

The presence of a T_23_ minimum, as function of temperature, indicates that proton relaxation measurements of water in such a system can be interpreted based on an ongoing chemical exchange process between water protons and hydrogen present on the hydroxyl groups of the polymer unit side chains. 

#### 3.1.2. Collagen Fibers as Affected by pH and Ionic Strength

**Figure 2:**
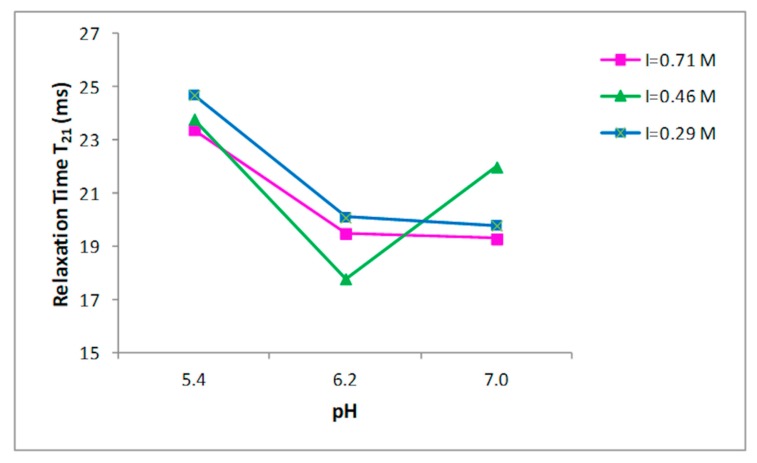
Relationship between the pH and T_21_ relaxation time in collagen at different ionic strengths.

**Figure 3:**
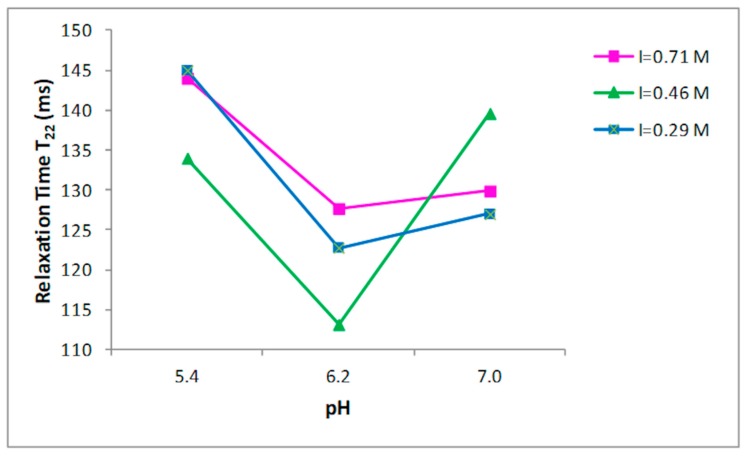
Relationship between the pH and T_22_ relaxation time in collagen at different ionic strengths.

**Figure 4:**
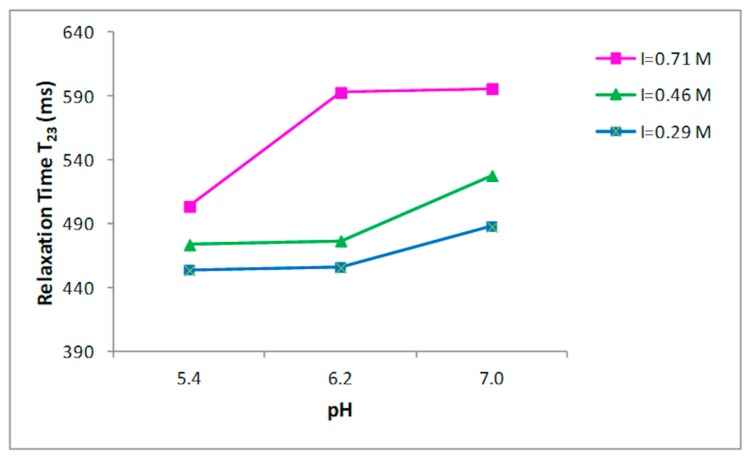
Relationship between the pH and T_23_ relaxation time in collagen at different ionic strengths.

Nine buffer solutions at three pH values, 5.4, 6.2, and 7.0, were prepared at three different ionic strengths: 0.29, 0.45, 0.71 M, as indicated in the Materials and Methods section. Prior to the 1H NMR measurements, the collagen fibers were hydrated by adding the buffer to the dried sample and left for two days at 4 °C. T_2_ relaxation times of hydrated collagen showed a triexponential decay with all buffer systems studied. T_21_, T_22_, and T_23_ relaxation times at the three ionic strengths as a function of pH are shown in Figure 2, Figure 3 and Figure 4, respectively. Both the ionic strength and pH affects significantly the T_2_ relaxation times. Such behavior was displayed by each of the three ionic strengths studied solutions.

Both the ionic strength and pH are reported to influence the water-holding capacity [29,30,31]. This behavior has been attributed to the effect of the pH on the net charge of the proteins, which determines the amount of water in these structures [32]. A strong effect of both the ionic strength and the pH on the distribution of T_2_ relaxation times was observed as reported in Figure 2, Figure 3 and Figure 4. 

Significant increases in the T_23_ relaxation times, which correspond to the “free” or “bulk” water fraction, with the increase of the ionic strength and pH were reported. Previous works, by investigating the effect of increasing the ionic strength in myofibrils by means of ^1^H NMR T_2_ relaxation measurements, showed how T_2_ relaxation times increase in accordance with the ionic strength [14,33,34]. The swelling of the collagen lattice with the increase of pH and ionic strength can well explain the obtained data [14,33,35].

These results confirm that the influence of pH changes on the fibrillar proteins’ net charge, and consequently, filament spacing is largest around the pK—that is, around pH~5 for the myofibrillar proteins; this support that the changes in relaxation time are related with the collagen lattice spacing, as underlined in the work by Offer and Trinick [30]. Several studies reported how T_2_ relaxation measured in muscle-based foods correlates with the total water content [36,37] and how T_2_ relaxation times are influenced by the ionic strength and pH. Noticeably, the effect of increasing pH from 5.4 to 6.2 had higher impact on the T_23_ relaxation than of increasing pH from 6.2 to 7.0 (Figure 4) when ionic strength was 0.71 M. An evident effect of the ionic strength on T_23_ relaxation times was observed at pH 6.2, when the ionic strength varies from 0.46 to 0.71 M. This indicates a correlation between ionic strength and the degree of fibrillar swelling. Indeed, a major change in the T_2_ relaxation times at pH 7.0 was observed when the ionic strength was respectively 0.29 and 0.46 M respect to the increase showed at 0.71 M (Figure 4). 

The myofibrillar swelling resulting from salting could be described hence by two main mechanisms, as indicated by Offer and Trinick [30]; in particular:

(i) the bond of negatively charged ions and the increase of electrostatic repulsion between the filaments, and

(ii) the removal of one or more transverse structural constraints in the structure, allowing the filament lattice to expand. 

Since the mechanism involving the removal of structural constraints in the collagen structure is a significative element for the swelling, it is possible to hypothesize that an upper level for the degree of swelling exists and that “saturation” is achieved in the ionic strength range between 0.46 and 0.71 M (Figure 4).

### 3.2. Characterization of Type I Collagen Modified with Catechin.

The water properties of collagen fiber systems modified with catechin were characterized using ^1^H NMR T_2_ relaxation measurements.

**Figure 5:**
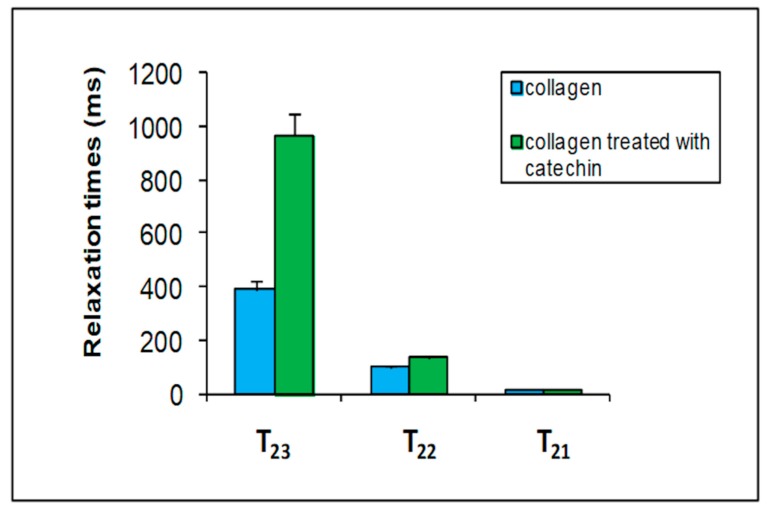
A comparison among the three transverse relation time (T_2_) components of collagen (blue columns) with those of collagen modified with catechin 0.01 M (green columns).

The CPMG decay of data from hydrated-modified collagen samples were multicomponent, as expected. Additionally, in this case, the sum of three exponentials led to the best fit. The three T_2_ values thus obtained were compared with those found for the collagen fibers (see Table 1) and are shown in Figure 5.

Catechin strongly affects the T_22_ and T_23_ water relaxation times of collagen. As mentioned before, the intermediate T_22_ was attributed to the water fraction in the interfibrillar space, and the third fraction (T_23_) corresponded to the free water fraction. These results indicate that catechin has been able to interact with collagen both on the fibrillar surface and inside the interfibrillar space. Water T_23_ relaxation value increases when the catechin is bound to the surface of collagen. This result could indicate that water interacts less with the surface of the polymer due to the formation of a complex between the catechin and collagen. 

## 4. Conclusions

The present study demonstrates that ^1^H NMR TD can be used to study the water dynamics of collagen thanks to its own longitudinal relaxation processes (T_1_ or spin-lattice) and transversal relaxation (T_2_ or spin-spin). Therefore, this approach could improve the understanding of how meat-processing factors can affect collagen swelling [38]. The spin-spin relaxation times reflect the molecular mobility of the collagen chain in accordance with changes in its own chemical structure also affecting meat quality traits related to the pH and ionic strength. Furthermore, the spin-spin relaxation time component T_23_ can be correlated to the WHC of the collagen-hydrated molecule. In fact, the composition and distribution of T_23_ fully represents the WHC: the higher the free water percentage, the lower the water retained inside the polymer and, consequently, also the WHC.

NMR relaxometry also allowed to investigate the interaction between collagen and catechin, an antioxidant molecule used in meat processing. In a previous work [7], it has been shown, from mono- and bi-dimensional proton NMR and ^13^C cross polarization magic angle spectroscopy experiments, that collagen-catechin interactions preferentially occur between the catechin B ring and the collagen proline and hydroxyproline amino acids. The results reported in this study can give further information on the interaction between catechin and collagen, indicating that catechin is able to bind collagen both on the fibrillar surface and inside the interfibrillar spaces of the polymer, giving a deeper insight in the understanding of the interaction between catechin and collagen.

## Figures and Tables

**Table 1:** Transverse relaxation time (T_2_i), population (fi), and diffusion coefficient (Di) values (mean ± standard deviation) related to each water fraction i obtained for hydrated collagen.

	Fraction i		
	1	2	3
T_2_i (ms)	6 ± 1	41 ± 7	447 ± 15
fi (%)	56 ± 5	36 ± 4	8 ± 2
Di (10^−5^cm^2^s^−1^)	0.71	1.22	2.31
